# C-754-05. Investigating Peripheral Cutaneous Innervation in Post-burn Wounds and Scars: Better Understanding Pain and Itch

**DOI:** 10.1093/jbcr/irag033.033

**Published:** 2026-04-06

**Authors:** Casey Meretta, Lian Daniel N Manalo, Kai V Myree Lindsey, Kenji Takamura, Donald Simone, Alyson C Farrer, Jeffrey W Shupp, Taryn E Travis, Phillip Rivera, Bonnie C Carney

**Affiliations:** The Burn Center at MedStar Washington Hospital Center, Washington, DC; The Burn Center at MedStar Washington Hospital Center, Washington, DC; The Burn Center at MedStar Washington Hospital Center, Washington, DC; University of Minnesota, Minneapolis, MN; University of Minnesota, Minneapolis, MN; Macalester College, Saint Paul, MN; MedStar Washington Hospital Center, Washington, DC; The Burn Center at MedStar Washington Hospital Center, Washington, DC; Macalester College, Saint Paul, MN; The Burn Center at MedStar Washington Hospital Center, Washington, DC

## Abstract

**Introduction:**

Burn injuries trigger long-lasting alterations in cutaneous innervation that contribute to chronic pain, itch, and sensory dysfunction. Epidermal nerve fiber density (ENFD) is a key biomarker of neural integrity, while vasoactive intestinal peptide (VIP) is a neuropeptide with potent effects on vasodilation, immune modulation, and wound healing. VIP upregulation in damaged skin may actively shape the wound environment by acting as a key ligand for paracrine signaling, influencing both nerve repair and inflammatory response cellular transduction pathways. Despite its potential importance, the coordinated analysis of ENFD and VIP expression in post-burn tissue has been limited by inconsistent methodology for their quantification.

**Methods:**

Deep partial thickness burn wounds were created in porcine skin (n = 6). On day 13, healed wound and adjacent normal skin biopsies were taken. Biopsies were fixed, cryosectioned at 60 μm, and immunostained for pan-neuronal marker protein gene product 9.5 (PGP9.5), VIP, and DAPI. The thick, 60 μm sections allowed for 3D evaluation of nerves that would not otherwise be captured by thin (6 μm) sections. Laser confocal microscopy was used to acquire 40 μm z-stacks at 2 μm intervals with a 10× objective. ENFs were traced using NeuronJ software, applying standardized rules for counting fibers crossing the dermal-epidermal junction. This approach included using the 3D data captured from z-stacking. Statistical testing was completed with Welch’s adjusted T.

**Results:**

Mean ENFD in normal skin was 10.36 ± 4.145 fibers/mm epidermis, compared with 2.489 ± 1.638 in the wound group, representing a 75.98% reduction (*p*=.0041). Representative confocal images confirmed consistent labeling of ENFs and clear delineation of the basement membrane. Inter-rater reproducibility of tracing was 0.920. VIP mean intensity values in normal skin were 11.16 ± 3.002 fluorescent intensity units (FIUs), compared with 26.61 ± 14.44 FIUs in the wounds, a 138.44% increase (*p*=.0465).

**Conclusions:**

This study demonstrates that healed burn wound is characterized by ENF loss and VIP upregulation. The findings suggest that VIP is not merely a byproduct of injury but may play a mechanistic role in modulating neural regeneration and inflammatory states after burn injury. By combining structural and molecular analyses, this methodology provides a new approach to investigate the relation between innervation and neuropeptide signaling in healing skin.

**Applicability of Research to Practice:**

Reduced ENFD provides an objective marker of small fiber loss, while VIP upregulation identifies a candidate therapeutic target for restoring balance between neural injury and repair. These measures could also be used to determine patients at risk for chronic pain and itch, guide neuro-regenerative treatment strategies, and monitor therapeutic efficacy over time.

**Funding for the study:**

This work was funded in part by award number KL2TR001432 from the National Center for Advancing Translational Science (NCATS/NIH).

